# Survey on the Impact of COVID-19 on the Practice of Assisted Reproduction in Latin America

**DOI:** 10.5935/1518-0557.20230035

**Published:** 2023

**Authors:** Maria do Carmo Borges de Souza, Natalia Posada, A.Gustavo Martinez, Adelino Amaral Silva, José Maria Mojarra Estrada, Diego Masoli, Fernando Zegers Hochschild, On behalf of REDLARA Member Centres

**Affiliations:** 1 Fertipraxis Centro de Reproducao Humana. Rio de Janeiro, RJ, Brazil; 2 Instituto Antioqueño de Reproducción. Medellin, Colombia; 3 Medicina Reproductiva Fertilis. Buenos Aires, Argentina; 4 Genesis-Centro de Assistencia em Reproducao Humana. Brasília, DF, Brazil; 5 Hospital CIMA, Hermosillo, Sonora, Mexico; 6 Clínica Las Condes Metropolitana de Santiago, Chile; 7 Programa de Etica y Politicas Publicas en Reproducción Humana, Facultad de Medicina Universidad Diego Portales. Director del Registro Latinoamericano de Reproducción Asistida, Chile

**Keywords:** SARS-CoV-2, COVID-19, pandemic, REDLARA Survey, Latin America

## Abstract

**Objective:**

To follow the impact of the SARS-CoV-2 pandemic on the practice of assisted reproductive technology in centers reporting to the Latin American Registry during 2020.

**Methods:**

An internally validated online survey designed on the Survey Monkey platform with a maximum of 20 closed questions was sent via e-mail or WhatsApp to the clinical director of each center reporting to the Latin American Registry of Assisted Reproduction between July and December 2020.

**Results:**

The number of centers responding to the survey varied during the six months. The relative contribution of Brazil to all responses was 41.4% to 45%, followed by México (16.2% to 23.8%), Argentina (8.1% to 12.6%), Colombia (7.1% to 8.2%), Chile (3.6% to 6.1%) and Peru (4.0% to 4.9%). Most centers reported stopping activities before July 2020 (81%). COVID-19 related symptoms were a criterion on their own to postpone ovarian stimulation (80.1% to 87.7% of centers). Although in July only 76 of 166 centers (45.8%) performed embryo transfers, by October 104 of 109 centers (95.4%) performed them. In survey 6 (December), 78 of 79 centers (98.7%) that had initially closed had already reopened, although 62.3% (61 of 98 centers) still performed 80% or less of their usual number of ART cycles.

**Conclusions:**

Most centers modified their clinical practice and applied specific protocols to screen their staff and patients. Suspicion of COVID-19 delayed treatments. Despite a peak of the pandemic, by December most centers were performing all ART treatments, although the number of cycles remained low compared to pre-pandemic numbers.

## INTRODUCTION

On December 31, 2019, twenty-seven cases of pneumonia of unknown cause originated in a seafood market in Wuhan, China, were officially notified (Mahase, 2020). The viral infection was reported to be due to a novel coronavirus, named severe acute respiratory syndrome coronavirus 2 (SARS-CoV-2) (Wang & Jin, 2020). Soon after, on January 30, 2020, the World Health Organization (WHO) declared this outbreak a “public health emergency of international concern” (WHO, 2020a), and on March 11, 2020, the Director-General of the WHO declared COVID-19 a global pandemic (WHO, 2020b). In the United States, the first cases of SARS-CoV-2 were reported on January 21, 2020, followed by France on January 25, 2020. In a 3-week period, between February and March, all 16 Latin American countries that report to REDLARA had verified COVID-19 cases (Souza *et al*., 2020). By June 2020, Latin America had become an important epicenter of the COVID-19 pandemic and Brazil, Peru, Chile and Mexico were among the most heavily affected; Brazil was second only to the United States in the number of reported cases (Worldometer, 2023).

The pandemic impacted all aspects of life with important economic and psychological consequences. Traditionally, practice guidelines are based on strong scientific evidence. However, in the case of COVID-19, medical societies have been forced to release and frequently modify position statements based on expert consensus or preliminary studies (ESHRE COVID-19 Working Group, 2020; Pirjani *et al*., 2020).

In the earliest stages of the pandemic, the American Society for Reproductive Medicine (ASRM) and the European Society of Human Reproduction and Embryology (ESHRE), independently recommended the discontinuation of reproductive care, except for the most urgent cases (Veiga *et al*., 2020). In response to the outbreak of the virus in Europe, the ESHRE released, on February 27, 2020, a first statement recommending avoiding ART pregnancies in patients with COVID-19, expanding it, two weeks later, to include all fertility patients. The ESHRE COVID-19 Working Group was then created to monitor and inform its members on the development of the pandemic. On April 2, 2020, the ESHRE advised not to start any ART cycles, except for the urgent preservation of gametes or germinal tissue, arguing the need to avoid potential SARS-CoV-2-related complications during treatment or pregnancy, mitigate the unknown risk of vertical transmission in SARS-CoV-2-positive patients, support the necessary re-allocation of healthcare resources, and to observe current social distancing recommendations (ESHRE, 2020). The ASRM assembled a COVID-19 Task Force and released an official document on March 17, 2020 (ASRM COVID-19 Task Force, 2020a), updated on March 30 (ASRM COVID-19 Task Force, 2020b), suggesting that all new treatments and embryo transfers should be suspended, treatment for patients already in the cycle or requiring urgent stimulation and cryopreservation continued, and elective surgery and non-urgent diagnostic procedures suspended, with an increase in adoption of telemedicine (Veiga *et al*., 2020).

In April 2020, after successful mitigation strategies and emergence of additional data, the ASRM, the ESHRE and The International Federation of Fertility Societies (IFFS) sanctioned a gradual and judicious resumption of reproductive care (Veiga *et al*., 2020; ESHRE COVID-19 Working Group, 2020; Practice Committee of the American Society for Reproductive Medicine, 2020), while promoting COVID-19-related research efforts that included the ASPIRE (Assessing the Safety of Pregnancy in the Coronavirus Pandemic) cohort study in the United States, between March 1 and December 31, 2020; the ESHRE global case-by-case report on the outcome of MAR conceived pregnancies in women with a confirmed infection (https://nl.surveymonkey.com/r/COVID19ART); the inclusion of mandatory COVID-19-related questions in the Clinic Outcome Reporting System Registry of ART cycles of the Society for Assisted Reproductive Technologies (SART) in the United States. The IFFS conducted COVID-19-related periodic surveys to assess global trends in access to MAR/ART services. Finally, the ESHRE devised a survey to measure the impact of the COVID-19 pandemic on the practice of MAR/ART across Europe, which allowed them to construct a timeline to supervise the discontinuation and restart of MAR/ART activities in Europe at 2-week intervals between March 1 and May 29, 2020, aligned to national data on daily reported COVID-19 cases and deaths.

Likewise, in March 17, 2020, the Brazilian Society of Assisted Reproduction and REDLARA published a first joint note urging centers to stop new ART procedures, with the exception of oncological or other cases where postponement would cause more harm to the patient (SBRA & REDLARA, 2020) This recommendation was modified in April 15, 2020, advising that ART cycles be carried out at the discretion of the attending physician, according to local government regulations, with embryo transfers where possible (Souza, 2020; Souza *et al*., 2020).

The primary objective of this survey was to evaluate the impact of the SARS-CoV-2 pandemic on the practice of reproductive medicine in REDLARA member centers according to country of origin and in consonance with governmental regulations and medical societies’ recommendations. A secondary objective was to describe the COVID-19-related biosecurity protocols implemented by the member centers and the prevalence of COVID-19 among patients and clinical staff.

For administrative purposes, REDLARA member centers are divided into four regions according to country of origin: Brazil, Mexico, Southern Cone Region (Argentina, Bolivia, Chile, Uruguay, and Paraguay) and the Northwestern Region (Colombia, Costa Rica, Ecuador, Guatemala, Nicaragua, Peru, Dominican Republic and Venezuela).

## MATERIAL AND METHODS

An online survey was designed on the Survey Monkey platform that included a maximum of 20 closed questions. Internal validation of the survey reported a maximum response time of 4 minutes. The survey was sent via e-mail or WhatsApp to the clinical director of each center reporting to the Latin American registry of ART (REDLARA), every month for six consecutive months, corresponding to surveys 1 to 6. In case of no response, the survey was also sent to the Laboratory Director. In accordance with confidentiality principles of the REDLARA registry, participating centers were anonymized. The survey included the country of origin of each member center, followed by questions with simple yes or no or multiple-choice answers related to the center’s current clinical practice, biosecurity protocols, and type and number of reproductive treatments performed during the six-month period. The responses were monitored by the REDLARA Regional Directors, representing all participating countries. In case of no response, a reminder was sent within up to seven days to the addressee. A response time of 15 days was provided for each questionnaire. A total of 197 centers were contacted.

## RESULTS

The survey was sent monthly between July and December 2020. The response rate by country throughout the study period is described in Table 1A. In general, there was variability in the number of centers that responded to the survey in each country during the six months of the study.

**Table 1A:** Response rate by country throughout the study period.

No. of centers contacted per Country	July	August	September	October	November	December
**Brazil** n=70	70 (100.0)	68 (97.1)	66 (94.3)	48 (68.6)	56 (80.0)	45 (64.3)
**Mexico** n=38	31 (81.6)	32 (84.2)	30 (79.0)	18 (47.4)	32 (84.2)	19 (5.0)
**Southern Cone Region** Argentina n=30 Chile n=10 Paraguay n=1 Bolivia n=3 Uruguay n=2	17 (33.3) 9 (90.0) 1 (100.0) 2 (66.7) 2 (100.0)	20 (66.7) 8 (80.0) 1 (100) 2 (66.7) 2 (100.0)	12 (40.0) 9 (90.0) 1 (100) 2 (66.7) 2 (100.0)	14(46.7) 4 (40.0) 1 (100) 2 (66.7) 2 (100.0)	13 (43.3) 7 (70.0) 1 (100) 1 (33.3) 0	8 (26.7) 7 (70.0) 0 1 (33.3) 2 (100.0)
**Northwest Region** Colombia n=12 Peru n=12 Venezuela n=6 Ecuador n= 6 Guatemala n=1 Nicaragua n=1 Panama n=3 Dominican Republic n=2	12 (100.0) 8 (66.7) 5 (83.3) 3 (50.0) 1 (100) 1 (100) 1 (33.3) 1 (50.0)	12 (100.0) 8 (66.7) 3 (50.0) 3 (50.0) 1 (100) 0 3 (100.0) 1 (50.0)	9 (75.0) 7 (59.3) 1 (16.7) 3 (50.0) 1 (100) 0 4 (133.0) 1 (50.0)	9 (75.0) 5 (41.7) 1 (16.7) 5 (83.3) 1 (100) 0 1 (33.3) 0	11 (91.7) 6 (50.0) 2 (33.3) 2 (33.3) 0 1 (100) 2 (66.7) 0	7 (59.3) 4 (33.3) 0 4 (66.7) 1 (100) 0 1 (33.3) 0

(%) corresponds to the proportion of centers that responded the survey each month.

The relative contribution of each country’s responses in surveys 1 to 6 is illustrated in Table 1B. Due to a variable number of responding centers throughout the study, this is illustrated as a range. Brazil contributed 41.4% to 45% of all responses, followed by México (16.2% to 23.8%), Argentina (8.1% to 12.6%), Colombia (7.1% to 8.2%), Chile (3.6% to 6.1%) and Peru (4.0% to 4.9%).

**Table 1B:** Response rate by country throughout the study period.

	July n=164	August n=164	September n=148	October n=111	November n=134	December n=99
**Brazil Region** 70	70 (42.6)	68 (41.5)	66 (44.6)	48 (43.2)	56 (41.8)	45 (45.5)
**Mexico Region** 38	31 (18.9)	32 (19.5)	30 (20.2)	18 (16.2)	32 (23.8)	19 (19.2)
**Southern Cone Region** Argentina 30 Chile 10 Paraguay 1 Bolivia 3 Uruguay 2	17 (10.4) 9 (5.4) 1 (0.6) 2 (1.2) 2 (1.2)	20 (12.2) 8 (4.9) 1 (0.6) 2 (1.2) 2 (1.2)	12 (8.1) 9 (6.1) 2 (1.3) 2 (1.3) 2 (1.3)	14 (12.6) 4 (3.6) 1 (0.9) 2 (1.8) 2 (1.8)	13 (9.7) 7 (5.2) 1 (0.7) 1 (0.7) 0	8 (8.1) 7 (7.1) 0 1 (1.0) 2 (2.0)
**Northwest Region** Colombia 12 Peru 12 Venezuela 6 Ecuador 6 Guatemala 1 Nicaragua 1 Panama3 Dominican Republic 2	12 (7.3) 8 (4.9) 5 (3.0) 3 (1.8) 1 (0.7) 1 (0.7) 1 (0.7) 1 (0.7)	12 (7.3) 8 (4.9) 3 (1.8) 3 (1.8) 1 (0.6) 0 3 (1.8) 1 (0.6)	9 (6.1) 7 (4.7) 1 (0.7) 3 (2.0) 1 (0.6) 0 3 (2.0) 1 (0.7)	9 (8.1) 5 (4.5) 1 (0.9) 5 (4.5) 1 (0.9) 0 1 (0.9) 0	11 (8.2) 6 (4.5) 2 (1.5) 2 (1.5) 0 1 (0.7) 2 (1.5) 0	7 (7.1) 4 (4.0) 0 4 (4.0) 1 (1.0) 0 1 (1.0) 0

(%) corresponds to the proportion of responses by each country relative to all participating countries.

To the question of whether the center had stopped activities due to the pandemic, 133 out of 164 centers (81.1%) responded stopping activities before survey 1 in July 2020. In the second survey, 139 out of 156 (89.1%) centers that closed had reopened for a period ranging from less than 30 days up to 60 days. The number of responding centers in surveys 3, 4, 5 and 6 was 148, 111, 134 and 99, respectively. Survey 6 reported that by December 2020, 69 out of 79 (87%) centers had reopened for over 60 days and only one center remained closed (Table 2).

**Table 2:** Response rate by country throughout the study period.

	July n=164	August n=164	September n=148	October n=111	November n=134	December n=99
**Closed**	133 (81.1)	156 (95.1)	116 (78.4)	88 (79.3)	114 (85.1)	79 (79.8)
**Not-reopened**		17 (10.4)	8 (5.4)	3 (2.7)	2 (1.5)	1 (1.0)
**Reopened centers** <30 days 31-45 days >46-60 days >61 days		47 (28.7) 54 (32.9) 38 (23.2)	5 (3.4) 32 (21.6) 40 (27.0) 32 (21.6)	4 (3.6) 4 (3.6) 12 (10.8) 65 (58.6)	6 (4.5) 4 (3.0) 16 (11.9) 86 (64.2)	1 (1.0) 5 (5.1) 3 (3.0) 69 (69.7)

**Figure 1:**
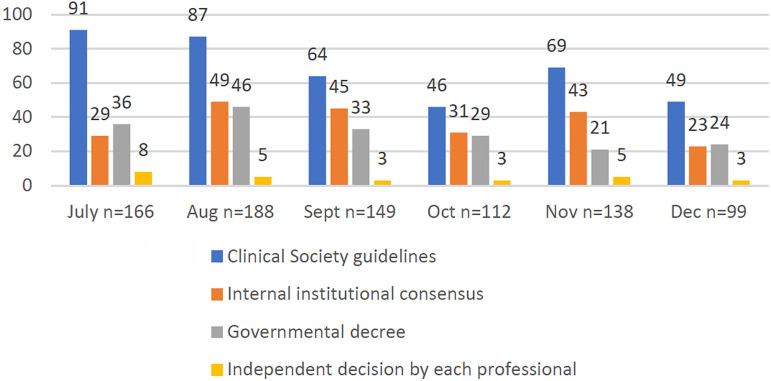
Distribution of responding centers according to criteria used to stop and/or resume reproductive treatments.

The main criteria used by the centers to suspend and/or restart reproductive treatments (Figure 1) were guidelines from medical societies, followed by institutional medical consensus, and government decrees.

**Figure 2:**
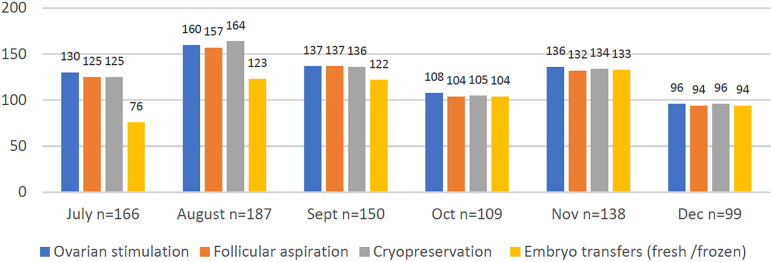
Distribution of centers according to the number of ART related procedures performed between July and December 2020.

**Figure 3:**
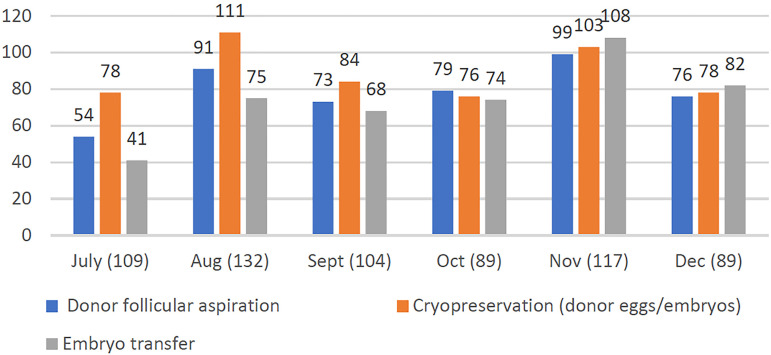
Distribution of centers with an oocyte donation program according to the number of donor egg-related procedures performed between July and December 2020.

With regards to the overall practice of ART (Figure 2), in July 2020, of 166 centers, 78.3% performed ovarian stimulations, 75.3% oocyte retrievals and cryopreservation of gametes or embryos, and 45.8% fresh or frozen embryo transfers. By September 2020, survey 3 reported that 137 of 150 centers (91.3%) were performing ovarian stimulations and oocyte retrievals, 136 (90.7%) cryopreserved embryos or gametes, and 122 (81.3%) were then performing embryo transfers. The number of centers performing embryo transfers increased to 95.4% (104 of 109 centers) in October and remained equally high during surveys 5 and 6. When inquired about oocyte donation, in July 2020, of 109 centers with an existing oocyte donation program, 54 (49.5%) performed donor follicular aspirations, 78 (71.6%) donor egg or embryo cryopreservation, and 41 (37.6%) donor egg embryo transfers. The number of donor egg embryo transfers increased every month, and in survey 6 (December), 82 of 89 (92.1%) centers were carrying out donor egg embryo transfers (Figure 3).

In surveys 1 to 6, 96.2% to 99.3% of the centers screened patients for symptoms or risk factors associated with COVID-19 before starting a treatment cycle (Table 3). Besides a confirmed diagnosis of COVID-19, the presence of related symptoms was, by itself, a criterion to postpone treatment in 80.1% to 85.7% of centers, followed by a history of a recent close contact (less than 14 days) with someone suspected of having the disease (69.4% to 73.5%). The percentage of centers that routinely requested a negative nasopharyngeal PCR swab (with or without antibodies against COVID-19) before the start of treatment ranged from 23.9% in July to 56.2% in November (Table 4). Likewise, the number of centers that routinely performed diagnostic tests on their clinical staff remained rather stable throughout the survey and ranged between 31.6% and 42.2% between July and December, except in the month of October, where only 13.6% of the centers reported screening their staff (Table 4).

**Table 3:** Distribution of centers (%) according to the use of screening for COVID-19 risk and criteria used to postpone treatment cycles.

	July n=159	Aug n=183	Sept n=147	Oct n=110	Nov n=137	Dec n=98
**No triage**	6 (3.8)	4 (2.2)	1 (0.7)	1 (0.9)	2 (1.5)	2 (2.0)
**Use of triage**	153 (96.2)	179 (97.8)	146 (99.3)	109 (99.1)	135 (98.5)	96 (98.0)
**Type of exclusion criteria used** -COVID 19 diagnosis -Recent travel by plane -Direct contact with a proven or suspected case of COVID-19 -Dry cough, fatigue, chest pain, diarrhea, skin rash, headache -Fever	141 (84.9) 89 (53.6) 118 (71.1) 133 (80.1) 120 (72.3)	159 (87.8) 93 (50.8) 127 (69.3) 155 (84.6) 141 (77)	139 (94.5) 79 (53.74) 108 (73.4) 129 (87.7) 120 (81.6)	103 (93.6) 48 (43.6) 74 (67.2) 94 (85.4) 80 (72.7)	129 (94.1) 56 (40.8) 96 (70) 113 (82.4) 103 (75.1)	92 (93.8) 33 (33.6) 63 (64.2) 84 (85.7) 78 (79.5)

**Table 4:** Number (%) of centers routinely performing diagnostic tests on their clinical staff or patients before the start of ovarian stimulation.

	July n=155	Aug n=181	Sept n=147	Oct n=110	Nov n=137	Dec n=98
Patients -Antibody tests (IgG /IgM) only -PCR nasopharynx swab	24 (15.5) 37 (23.9)	38 (21.0) 60 (33.2)	51 (46.3) 72 (49.0)	31 (28.1) 55 (50.0)	43 (31.15) 77 (56.2)	27 (27.55) 50 (51.0)
Clinical Staff -PCR nasopharynx swab and/or antibodies (IgG and IgM)	49 (31.6)	75 (41.4)	62 (42.2)	15 (13.6)	53 (38.71)	35 (35.7)

The proportion of centers reporting patients with suspected or confirmed COVID-19 increased from 26.3% (47 of 179 centers) in August to 66.3% (65 of 98 centers) in December 2020. (Table 5). However, most centers had 10% or less of their patients affected with COVID-19 in surveys 1 to 6. Similarly, the number of centers with suspected or confirmed COVID-19 among their clinical staff increased from 30.6% (55 of 180 centers) in August to 61.4% (62 of 101 centers) in December. In this month, 17.5% (17 of 97 centers) reported having more than 10% of their clinical staff affected with COVID-19 (Table 6).

**Table 5:** Number (%) of centers routinely performing diagnostic tests on their clinical staff or patients before the start of ovarian stimulation.

Patients with positive symptoms or COVID 19 diagnostic test	July	Aug n=179	Sept n=144	Oct n=110	Nov n=138	Dec n=98
**No cases**		132 (73.7)	91 (63.2)	52 (47.3)	55 (39.9)	33 (33.7)
**Positive cases**		47 (26.3)	53 (36.8)	58 (52.7)	83 (60.1)	65 (66.3)
**Proportions of affected patients** 1-2% 3-5% 6-10% >11%		28 (15.6) 9 (5.0) 7 (3.9) 3 (1.7)	30 (20.8) 15 (10.4) 8 (5.6) 0	32 (29.1) 14 (12.7) 11 (10.0) 1 (0.9)	50 (36.2) 22 (15.9) 10 (7.2) 1 (0.7)	33 (33.7) 24 (24.5) 6 (6.1) 2 (2.0)

**Table 6:** Number (%) of centers routinely performing diagnostic tests on their clinical staff or patients before the start of ovarian stimulation.

	July	August n=180	Sept n=147	Oct n=110	Nov n=138	Dec n=99
No cases		125 (69.4)	91 (61.9)	57 (51.8)	63 (45.7)	39 (39.4)
Positive cases		55 (30.6)	56 (38.1)	53 (50.2)	75 (54.3)	60 (60.6)
Proportion of affected clinical staff 1-2% 3-5% 6-10% >11%		24 (13.3) 17 (19.4) 9 (5.0) 5 (2.8)	30 (20.4) 11 (7.5) 9 (6.1) 6 (4.0)	24 (21.8) 18 (16.4) 8 (7.3) 3 (2.7)	38 (27.5) 17 (12.3) 12 (8.7) 8 (5.7)	17 (17.5) 14 (14.4) 12 (12.4) 17 (17.5)

Given the consultation in surveys 1 to 6, between 7.1% to 13.6%, of the centers would continue to perform follicular aspirations in case of patients suspicious of or with confirmed COVID-19 during ovarian stimulation (Table 7). When asked about the fate of these oocytes (surveys 3 to 6), most of these centers would freeze all oocytes and or freeze all embryos in separate cryotanks. However, a minority of centers would cryopreserve gametes or embryos in routine cryotanks.

**Table 7:** Number of centers in which follicular aspiration was performed in case of a patient or couple with symptoms or confirmed COVID-19 during ovarian stimulation

	July n=155	August n=183	September n=147	October n=110	Nov n=137	Dec n=98
No	144 (92.9)	170 (92.9)	128 (87.1)	95 (86.4)	120 (87.6)	85 (86.7)
Yes	11 (7.1)	13 (7.1)	19 (12.9)	15 (13.6)	17 (12.4)	13 (13.3)
Oocyte freeze-all -in separate cryotank -in routine cryotank			17 5	10 4	13 5	12 4
Embryo freeze-all -in separate cryotank -in routine cryotank			7 4	2 4	10 4	2 2

Most centers gradually restarted activities during the second half of 2020. Table 8 distributes the centers according to the percentage of ART cycles performed during the six months of the survey compared to the months prior to the pandemic. In July 2020, 110 of 148 centers (74.3%) performed 20% or less of the usual number of ART cycles prior to the pandemic; the number of cycles gradually increased in surveys 2 to 6, and during the months of August, September, October, November, and December, the number of centers doing 20% or less of their usual cycles decreased to 42.4%, 31.1%, 19.1%, 15.2% and 9,2%, respectively. Nevertheless, in survey 6 (December) only 37 of 98 centers (37.7%) performed 80% or more of the number of ART cycles prior to the pandemic.

**Table 8:** Number (%) of responding centers according to the proportion of ART cycles performed during the 6-month survey compared to the pre-pandemic number of cycles.

Cycles	July n=148	Aug n=181	Sept n=148	Oct n=110	Nov n=138	Dec n=98
0-20	110 (74.3)	77 (42.5)	46 (31.1)	21 (19.1)	21 (15.2)	9 (9.2)
21-40	24 (16.2)	49 (27.1)	43 (29.1)	10 (9.1)	21 (15.2)	11 (11.2)
41-60	10 (6.7)	39 (21.5)	40 (27.0)	37 (33.6)	21 (15.2)	16 (16.3)
61-80	2 (1.4)	8 (4.4)	7 (4.7)	26 (11.8)	35 (25.4)	25 (25.5)
>80	2 (1.4)	8 (4.4)	12 (8.2)	16 (14.5)	40 (28.9)	37 (37.7)

## DISCUSSION

The first case of COVID-19 in Latin America was reported in Brazil on February 21, 2020, almost a month after the first COVID-19 cases had been reported in the United States and Europe (Rodriguez-Morales *et al*., 2020). On March 17, 2020, the Brazilian Society of Assisted Reproduction and REDLARA published a joint note recommending centers to stop any new non-urgent treatment cycles (SBRA & REDLARA, 2020). Despite institutional and governmental mitigation efforts, Latin America and the Caribbean witnessed an exponential increase in COVID-19 cases and deaths by mid-April 2020.

According to the ESHRE SARS-CoV-2 Work Group survey (ESHRE COVID-19 Working Group, 2020), Italy was the first country where MAR/ART activity was stopped on March 1, 2020. By March 15, 2020, nine European countries had discontinued all ART activity, in line with the exponential phase of the first COVID-19 epidemiologic curve and shortly after the WHO had declared COVID-19 a global pandemic and the ESHRE and other international scientific societies recommended postponing pregnancies through ART. The REDLARA survey was sent out in July 2020, three months after the initiation of the ESHRE SARS-CoV-2 Work Group survey and more than two months after ART activity in Europe had restarted in accordance with the ESHRE’s guidance for recommencing ART treatments on April 23, 2020 (ESHRE COVID-19 Working Group, 2021). Eighty one percent of REDLARA centers (133 of 164) reported stopping all activities before receiving this survey in July. Although the exact dates in which activities were stopped or resumed were not inquired, 139 out of 156 centers (89.1%) that had closed due to the pandemic, had reopened in August; fifty-two between 31 and 45 days; and 38 between 46 and 60 days. That is, 92 of 156 centers had resumed activities sometime in June or July, at a time when the highest numbers of new daily cases and deaths were reported from COVID-19 in Latin America and the Caribbean (100,057 and 3,070, respectively). This suggests that, contrary to European centers, the decision to stop and resume activities in Latin America did not follow the timeline of the peak of the first pandemic curve and was made more in line with the recommendations of international and regional scientific societies or governmental decrees. In fact, close to 50% of the centers stated that the decision to either stop or resume activities followed the guidelines of medical societies. Survey 6 reported that, in December 2020, all but one center (1 of 79) that had initially closed had now reopened, and almost all centers were performing embryo transfers at a time when Latin America and the Caribbean were in the exponential rise of the second peak of the pandemic (104,617 and 2,006 daily cases of infections and deaths from COVID-19, respectively). By January 2021, the number of new daily COVID-19 infections doubled the number of the first peak and Brazil had 6.9 million confirmed COVID-19 cases and 181,400 reported deaths. Resumption of ART in the region occurred before vaccines were available. The first regional vaccination programs began in Mexico, Chile, and Costa Rica on December 24, 2020, with Sinovac and the Pfizer vaccine, and in Argentina on December 29, 2020 with the Sputnik V vaccine (Lawton, 2021).

Although most centers restarted activities during the second half of 2020, the number of ART cycles remained quite low compared to the practice of ART before the pandemic. As of July 2020, two thirds of the centers reported performing 20% or less of their usual number of ART cycles. Although the number of cycles increased, in September more than 60.2% of the centers were still doing 40% or less of the usual number of cycles before the pandemic. In December, only 37.7% of centers were performing 80% or more of their usual number of ART cycles.

The proportion of centers performing routine PCR nasopharyngeal swabs either alone or in combination with IgM/IgG antibodies in triage-negative patients was low, and despite doubling during the six months of the survey, in December 2020, only 50% of the centers performed diagnostic tests in patients before starting treatment. The number of centers performing routine PCR nasopharyngeal swabs on their clinical staff was even lower (from 31.8% to 41.8%), despite an increase in the proportion of centers reporting patients and clinical staff with suspected or confirmed COVID-19 (from 22.3% and 31.6% in July to 66.3% and 57.7% in December 2020, respectively). The presence of COVID-19-related symptoms and history of a recent close contact (less than 14 days) with someone suspected of having the disease remained the principal criteria to delay the start of treatment.

The present report has some limitations, in that data was volunteered by the directors of centers affiliated with REDLARA. The number of centers responding the survey declined after August, and in December, only 99 centers responded compared to 164 in July. The reason for this decrease in the response rate might be that by November most centers had already adapted their practice to the pandemic, were performing all aspects of ART, and had no further changes to report. Because the name of the center was not necessarily included in the survey to protect its anonymity according to REDLARA’s confidentiality principles, it cannot be ruled out that some centers may have answered the survey twice (medical director and laboratory director), causing duplication of responses from the same center.

The SARS-CoV-2 pandemic impacted the practice of human reproduction throughout the World, and Latin America was no exception. This study offered a unique opportunity to gather information about the effects of COVID-19 in REDLARA member centers and their response to the pandemic. Most centers modified their clinical practice protocols and screened patients and staff to detect possible cases of COVID-19 and delayed treatment of patients with suspected or confirmed COVID-19. Despite the second peak of the pandemic, by December 2020, most centers were performing all aspects of ART, although the number of cycles remained low compared to pre-pandemic numbers.

### Abbreviations

REDLARA: Red Latinoamerica de Reproducción Asistida

SARS-CoV-2: severe acute respiratory syndrome coronavirus 2

MAR: medically assisted reproduction

ART: assisted reproduction treatment

## References

[r1] ASRM COVID-19 Task Force (2020a). Patient Management and Clinical Recommendations During The Coronavirus
(COVID-19) Pandemic.

[r2] ASRM COVID-19 Task Force (2020b). Patient Management and Clinical Recommendations During The Coronavirus
(COVID-19) Pandemic.

[r3] ESHRE - European Society of Human Reproduction and
Embryology (2020). A statement from ESHRE for phase 1 - Guidance on fertility services
during pandemic.

[r4] Gianaroli L, Ata B, Lundin K, Rautakallio-Hokkanen S, Tapanainen JS, Vermeulen N, Veiga A, Mocanu E., ESHRE COVID-19 Working Group (2021). The calm after the storm: re-starting ART treatments safely in
the wake of the COVID-19 pandemic. Hum Reprod.

[r5] Vermeulen N, Ata B, Gianaroli L, Lundin K, Mocanu E, Rautakallio-Hokkanen S, Tapanainen JS, Veiga A., ESHRE COVID-19 Working Group (2020). A picture of medically assisted reproduction activities during
the COVID-19 pandemic in Europe. Hum Reprod Open.

[r6] Lawton G. (2021). Sputnik V vaccine goes global. New Sci.

[r7] Mahase E. (2020). China coronavirus: what do we know so far?. BMJ.

[r8] Pirjani R, Rabiei M, Abiri A, Moini A. (2020). An Overview on Guidelines on COVID-19 Virus and Natural and
Assisted Reproductive Techniques Pregnancies. Int J Fertil Steril.

[r9] Practice Committee of the American Society for Reproductive
Medicine (2020). Recommendations for reducing the risk of viral transmission
during fertility treatment with the use of autologous gametes: a committee
opinion. Fertil Steril.

[r10] Rodriguez-Morales AJ, Gallego V, Escalera-Antezana JP, Méndez CA, Zambrano LI, Franco-Paredes C, Suárez JA, Rodriguez-Enciso HD, Balbin-Ramon GJ, Savio-Larriera E, Risquez A, Cimerman S. (2020). COVID-19 in Latin America: The implications of the first
confirmed case in Brazil. Travel Med Infect Dis.

[r11] Salgueiro L, Nakagawa H, Souza MC, Taitson P, SBRA - Associação Brasileira de
Reprodução Assistida; REDLARA - A Red Latinoamerica de
Reproducción Asistida (2020). Interfaces: Human Reproduction and COVID-19.

[r12] Souza MCB., Salgueiro L, Nakagawa H, Souza MC, Taitson P (2020). Interfaces: Human Reproduction and COVID-19.

[r13] Souza MCB, Nakagawa H, Taitson PF, Cordts EB, Antunes RA. (2020). Management of ART and COVID-19: Infertility in times of
pandemic. What now? JBRA Assist Reprod.

[r14] Veiga A, Gianaroli L, Ory S, Horton M, Feinberg E, Penzias A. (2020). Assisted reproduction and COVID-19: A joint statement of ASRM,
ESHRE and IFFS. Fertil Steril.

[r15] Wang G, Jin X. (2020). The progress of 2019 novel coronavirus event in
China. J Med Virol.

[r16] WHO - World Health Organization (2020a). Statement on the second meeting of the International Health Regulations
(2005) Emergency Committee regarding the outbreak of novel coronavirus
(2019-nCoV).

[r17] WHO - World Health Organization (2020b). WHO Director-General’s opening remarks at the media briefing on
COVID-19.

[r18] Worldometer COVID Live - Coronavirus Statistics.

